# A clinical prediction model for predicting the surgical site infection after an open reduction and internal fixation procedure considering the NHSN/SIR risk model: a multicenter case–control study

**DOI:** 10.3389/fsurg.2023.1189220

**Published:** 2023-09-19

**Authors:** Niloufar Taherpour, Yadollah Mehrabi, Arash Seifi, Seyed Saeed Hashemi Nazari

**Affiliations:** ^1^ Infectious Diseases and Tropical Medicine Research Center, Shahid Beheshti University of Medical Sciences, Tehran, Iran; ^2^ Department of Epidemiology, School of Public Health and Safety, Shahid Beheshti University of Medical Sciences, Tehran, Iran; ^3^ Department of Infectious Diseases, Faculty of Medicine, Tehran University of Medical Sciences, Tehran, Iran; ^4^ Prevention of Cardiovascular Disease Research Center, Department of Epidemiology, School of Public Health and Safety, Shahid Beheshti University of Medical Sciences, Tehran, Iran

**Keywords:** surgical site infection, ORIF surgery, prediction model, standardized infection ratio, nosocomial infection

## Abstract

**Introduction:**

Surgical site infection (SSI) is one of the most common surgical-related complications worldwide, particularly in developing countries. SSI is responsible for mortality, long hospitalization period, and a high economic burden.

**Method:**

This hospital-based case–control study was conducted in six educational hospitals in Tehran, Iran. A total of 244 patients at the age of 18–85 years who had undergone open reduction and internal fixation (ORIF) surgery were included in this study. Among the 244 patients, 122 patients who developed SSIs were selected to be compared with 122 non-infected patients used as controls. At the second stage, all patients (*n* = 350) who underwent ORIF surgery in a hospital were selected for an estimation of the standardized infection ratio (SIR). A logistic regression model was used for predicting the most important factors associated with the occurrence of SSIs. Finally, the performance of the ORIF prediction model was evaluated using discrimination and calibration indices. Data were analyzed using R.3.6.2 and STATA.14 software.

**Results:**

*Klebsiella* (14.75%) was the most frequently detected bacterium in SSIs following ORIF surgery. The results revealed that the most important factors associated with SSI following an ORIF procedure were found to be elder age, elective surgery, prolonged operation time, American Society of Anesthesiologists score of ≥2, class 3 and 4 wound, and preoperative blood glucose levels of >200 mg/dl; while preoperative higher hemoglobin level (g/dl) was found to be a protective factor. The evidence for the interaction effect between age and gender, body mass index and gender, and age and elective surgery were also observed. After assessing the internal validity of the model, the overall performance of the models was found to be good with an area under the curve of 95%. The SIR of SSI for ORIF surgery in the selected hospital was 0.66 among the patients aged 18–85 years old.

**Conclusion:**

New risk prediction models can help in detecting high-risk patients and monitoring the infection rate in hospitals based on their infection prevention and control programs. Physicians using prediction models can identify high-risk patients with these factors prior to ORIF procedure.

## Introduction

Surgical site infection (SSI) is one of the most common complications following surgery worldwide, particularly in the developing countries. In addition, SSI is the second most common type of nosocomial infections (NIs) (1). According to the report of Centers for Disease Control and Prevention (CDC), SSI is an infection that occurs within 30 days after general surgery or within a year after a surgery in which a non-human-derived implantable foreign body, such as prosthesis, artificial joint (in orthopedic surgery), or prosthetic heart valve, is implanted (2). In general, the prevalence rate of nosocomial infections related to SSI in Europe and North America is 5%, and the prevalence rate in the sub-Saharan Africa and Latin America and parts of Asia is 40%. Studies have also shown that the prevalence rate of nosocomial infections in Iran is estimated to be approximately 8%–10% (3). Despite the promotion of quality of healthcare services and employing new methods in preventing and controlling the infection, NIs are still a threatening risk in health centers, particularly in developing countries. The increasing rate of nosocomial infections during the past years was due to the following factors: antimicrobial resistance, invasive diagnostic and therapeutic techniques such as the use of catheter and ventilator, non-sterilization of surgical equipment, increase in the mean age of the population, patient characteristics such as weakening of the immune system, occurrence of various diseases such as diabetes, and hospital congestion due to patients suffering from chronic diseases and accidents (4). Although sanitation level of operating rooms, hygiene status of patients, and hospital personnel can be involved in the incidence of NIs, some intrinsic and non-modifiable factors may affect the occurrence of NIs, which can be minimized by recognizing and controlling them (5). Prediction models along with active surveillance systems can be helpful as tools in identifying the factors associated with the incidence of NIs. In fact, by identifying the relevant factors and estimating the probability of SSIs in each patient prior to surgery and the risk spectrum of the patients, it is possible to identify high-risk patients and take appropriate measures in preventing adverse effects (6). The National Healthcare Safety Network (NHSN) organization developed and updated, in 2015, the standardized infection ratio (SIR), which is an index to monitor the healthcare-associated infections (HAIs). In fact, the SIR index based on prediction models standardizes the NIs rate by considering environmental and individual factors (7). With regard to the high number of orthopedic surgeries in Iran that is accompanied by a great number of NIs, as well as the lack of active surveillance systems, the present study is designed to identify the risk factors related to SSI following an open reduction and internal fixation (ORIF) procedure according to the proposed model by NHSN and its application in standardizing the infection rate after an ORIF surgery at educational hospitals in Tehran.

## Material and methods

### Study design and patients

A multicenter hospital-based case–control study was conducted at six educational hospitals in Tehran, Iran. In this study, 244 adults (aged ≥18 years old) who had undergone an operative procedure of open reduction of fracture or dislocation of long bones that required internal or external fixation (ORIF) were included. This study was approved by the ethics committee of Shahid Beheshti University of Medical Sciences (SBMU), Tehran, Iran (IR.SBMU.RETECH.REC.1400.234).

In this study, the case group was defined as a person who received ORIF surgery and acquired surgical site infection within 1 year after the operative procedure. The control group included patients who underwent the same operative procedure but had no surgical site infection in the following year after the operation. A frequency matching method was performed with controls and cases according to the date of operation, ward of hospitalization, and type and site of surgery based on ICD-9-CM codes.

Detecting of surgical site infection was based on localized signs or symptoms and blood culture or non-culture-based microbiologic testing method or based on physician confirmation of the presence of infection. The inclusion criteria in this study were based on the criteria of the NHSN model “all SSI SIR model” that included only inpatient procedures, SSIs that are superficial, deep, and organ/space, and SSIs identified on admission and readmission. Any other types of orthopedic surgeries aside from ORIF such as placement of joint prosthesis were excluded from the study.

During the surgery, appropriate antibiotics were administered to target the most common organisms based on the standard preoperative antibiotic regimen. The international and national guidelines for administering of antimicrobial prophylaxis in orthopedic surgery are as follows (8, 9).

Cefazolin, 2 g (3 g for weight of >120 kg), was administered 30–60 min prior to surgery. Alternatively, vancomycin 15 mg/kg, 60 min prior to surgery, was administered to patients with history of beta-lactam allergy. For surgical durations of more than 4 h or for surgery cases with estimated blood loss over 1,500 ml, dosing of antibiotics was repeated. Administering of antibiotic continued 24 h postoperatively.

### Sample size and sample selection

In this study, 244 patients were included from six educational hospitals in Tehran, Iran. Six educational hospitals were selected from three medical sciences universities including SBMU from the north and east part of the province, Tehran University of Medical Sciences (TUMS) from the center and south part, and Iran University of Medical Sciences (IUMS) from the west part. All of the educational and general hospitals with more than 250 hospital bed capacity from each university were included in the sampling frame, and then three hospitals from each university were randomly selected. From the nine selected hospitals, only six hospitals were willing to cooperate in our research.

The sample size requirement for prediction models is often calculated according to the rule of thumb of “at least 10 Events Per Variable (EPV)” (10). Based on the number of collected predictors, a total of 244 patients were required (122 cases, 122 controls). The 244 patients included in this study were divided among the six selected hospitals. In fact, we selected approximately 40 patients (20 cases and 20 controls) from the list of total ORIF procedures in each hospital. For calculating the SIR in one of the hospitals, we randomly selected one hospital out of the six hospitals, and then all the patients who had undergone ORIF surgery in that hospital were extracted.

### Data gathering

The characteristics of patients were extracted from their medical records using a researcher-made checklist. We gathered data for the following variables: demographic characteristics, habits, Charlson comorbidity index (CCI) (11), type of hospital admission, length of hospital stay, time of procedure, procedure duration, vital status at discharge, laboratory test results prior to surgery, type of anesthesia used, catheterization and drainage, results of microbiologic test, administering of prophylaxis prior to surgery, and the suggested NHSN risk factors in 2018 for ORIF surgery such as the American Society of Anesthesiologists (ASA) score (12), wound class, closure technique, and body mass index (BMI).

### Statistical analysis

The normality of continuous variables was checked using *Q*–*Q* plot. Continuous variables were reported as means and standard deviations (SD) or median and interquartile range (*Q*1–*Q*3), and categorical variables were described as frequency and percentage. For comparison of means between two groups, Student's *t*-test and Mann–Whitney *U* test were used. For comparing categorical variables between groups, we used *χ*^2^ or Fisher's exact test whenever appropriate. All statistical analyses were conducted at a significance level of 0.05 using STATA version 14 and R software version 3.6.2.

### Prediction model development and intercept correction

Selecting of variables to develop a prediction model was conducted by employing a stepwise selection method with forward and backward approaches based on Akaike's information criterion (AIC) and using the “MASS” package. After selecting the best predictors of SSI, a multivariable logistic regression was used for developing of a model. Regression coefficients and crude and adjusted odds ratio (OR) were reported.

Given that the ratio of cases to controls was 1:1 and the actual incidence rate of SSIs following ORIF surgery in Tehran was approximately 8% during the period of 2017–2018 (based on incidence of SSIs following ORIF surgery among the research hospitals), the intercept of the final multivariable model was updated to account for this difference in the rate of infection between the data and the actual population according to the following formula (10, 13):

$$\mathrm{Corrected}\;\mathrm{Intercept}\;=\;\mathrm{Estimated}\;\mathrm{intercept}\;\;+\;\mathrm{Ln}\left[\frac{n_{\mathrm{controls}}}{n_{\mathrm{cases}}}\times \frac{\hat{\;p}}{1-\hat{\;p}}\right]$$

$$\mathrm{Corrected}\;\mathrm{intercept}\;\mathrm{based}\;\mathrm{on}\;\hat{\;p}(0.08)\;=\;0.7836\;+\;(-2.442347)\;=\;-1.6588$$

### Model performance and validation

The AIC criterion, Nagelkerke *R*^2^, Brier score (distance between the predicted and actual outcome), and scaled Brier score (corrected Brier score for the prevalence of SSI in the studied population) were used to evaluate the overall performance of the prediction model. The validity of the final model was assessed using validity indices such as accuracy, sensitivity, and specificity. The optimal cut point for dichotomizing the predicted probabilities was selected by maximizing the sensitivity and specificity. Discrimination slope using box plot, C-index, and area under the curve (AUC, ROC curve) were used to assess the discrimination ability of the model (10). The calibration of the model was checked based on Hosmer–Lemeshow test and calibration plot (comparing the predicted probability of SSI against observed proportion). The internal validity of the model was evaluated using a split-sample validation method. In this method, data are randomly divided into train (70% of data for constructing the prediction model) and test (30% of data for evaluating the validity of the prediction model), and then all of the overall performance, validation, and calibration indicators were calculated and compared.

### Standardized infection ratio

The SIR is estimated by dividing the number of observed SSIs by the number of predicted SSIs. The number of predicted SSIs was calculated using the selected logistic regression model, utilizing several factors that were found to be associated with the occurrence of SSIs. An SIR value greater than 1.0 means that the observed number of SSIs is more than what was predicted, and an SIR value of less than 1.0 shows that a smaller number of SSIs were observed than what was predicted (7). Using a multivariable logistic regression, we predicted the log-odds of SSIs following ORIF surgery. Then, the log-odds was converted into probability and summed to get the number of expected SSIs for the total data of one hospital (predicted SSIs). Finally, the SIR for ORIF surgery during the period of March 2017 to March 2018 in a hospital in Tehran was calculated as follows: actual number of reported SSIs following ORIF procedure during the specific time in a hospital (*O*) divided by the total number of predicted SSIs of the same procedure and time period (*P*).

The SIR estimation for ORIF procedure in one hospital was calculated as follows:

$$\mathrm{logit}\;(\;p_{i})=\;\alpha_{\mathrm{corrected}}+\beta_{1}x_{1}+\;\beta_{2}x_{2}+\ldots +\beta_{i}x_{i}$$

$$P=\frac{e^{\mathrm{logit}\;(\;p_{i})}}{1+\;e^{\mathrm{logit}\;(\;p_{i})}}\;\mathrm{SIR}=\;\frac{\mathrm{Observed}\;\mathrm{SSIs}\;(O)}{\mathrm{Predicted}\;\mathrm{SSIs}\;(P)}$$

## Results

### General information

The mean age of the 244 patients who underwent ORIF surgery was 43.72 ± 18.83 years old, of which 186 (76.23%) the patients were men. The ORIF surgeries were performed on the following sites: 89 (36.48%) patients were operated on the tibia and fibula, 80 (32.79%) patients were operated on the femur, and 28 (11.48%) patients were operated on the humerus, radius, and ulna. The median of hospital stay was 8 (6–13) days, and the length of hospitalization among infected patients was statistically longer than that of the non-infected patients (*P*-value < 0.001). The median time between surgery and SSI occurrence was 29.50 (14–61) days. Prophylaxis was administered correctly in 89.34% of the surgeries in this study based on the national guideline. The difference in administering of prophylaxis between the infected (90.98%) and non-infected (87.70%) groups was not statistically significant (*P-*value = 0.407). *Klebsiella* (14.75%), *Staphylococcus aureus* (13.11%), and *Enterobacter* (6.56%) were the most prevalent bacteria found in SSIs following ORIF procedure. Only three patients died in the hospital due to SSI following ORIF procedure. The details of demographic and clinical information of patients are reported in Table 1.

**Table 1:** Comparison of demographic and clinical characteristics of patients who underwent ORIF surgery between the infected and non-infected groups.

Variables	All patients (*n* = 244)	Infected (*n* = 122)	Non-infected (*n* = 122)	*P*-value
General characteristics				
Age (years)	43.72 ± 18.83	44.88 ± 17.62	42.57 ± 19.96	0.129
Sex				
Female	58 (23.77)	27 (22.13)	31 (25.41)	0.457
Male	186 (76.23)	95 (77.87)	91 (74.59)	
Past medical history				
BMI (kg/m^2)^	25.26 ± 3.94	25.94 ± 4.25	24.57 ± 3.48	0.017
Smoking (yes)	87 (35.66)	52 (42.62)	35 (28.69)	0.023
Charlson comorbidity index (CCI ≥ 1 score)	70 (28.69)	42 (34.43)	28 (22.95)	0.048
Hospitalization information				
Type of admission				
Elective	82 (33.61)	46 (37.70)	36 (29.51)	
Emergency	162 (66.39)	76 (62.30)	86 (70.49)	0.175
Duration of hospitalization (days)	8 (6–13)	11.50 (6–19)	7 (5–10)	<0.001
Time between surgery and infection (days)	—	29.50 (14–61)	N/A	—
Vital status				
Lived	241 (98.77)	119 (97.54)	122 (100)	0.247
Died	3 (1.23)	3 (2.46)	0 (0)	
Laboratory findings				
Hemoglobin (g/dl) last value prior to surgery	12.32 ± 2.14	11.80 ± 2.07	12.83 ± 2.10	0.0001
White blood cell count (WBC, 10^3^/L), last value prior to surgery	9.83 ± 3.27	9.63 ± 3.47	10.03 ± 3.06	0.143
Blood sugar (>200 mg/dl), last value prior to surgery	18 (7.38)	12 (9.84)	6 (4.92)	0.142
Information of pre and post procedure				
Duration of procedure (minutes)	186.42 ± 77.85	200.08 ± 82.05	172.77 ± 71.16	0.004
ASA score				
ASA 1	99 (40.57)	35 (28.69)	64 (52.64)	0.001
ASA 2	120 (49.18)	70 (57.38)	50 (40.98)	
ASA ≥ 3	25 (10.25)	17 (13.93)	8 (6.56)	
Type of anesthesia				
General	127 (52.05)	71 (58.20)	56 (45.90)	0.055
Spinal/epidural	117 (47.95)	51 (41.80)	66 (54.10)	
Wound class				
Class 1	157 (64.34)	61 (50.0)	96 (78.69)	0.001
Class 2	13 (5.33)	7 (5.74)	6 (4.92)	
Class 3	48 (19.67)	32 (26.23)	16 (13.11)	
Class 4	26 (10.66)	22 (18.03)	4 (3.28)	
Wound closure technique				
Primary	240 (98.36)	118 (96.72)	122 (100)	0.122
Secondary	4 (3.28)	4 (3.28)	0 (0)	
Administration of prophylaxis prior to surgery (yes)	218 (89.34)	111 (90.98)	107 (87.70)	0.407
Drainage (yes)	71 (29.10)	39 (31.97)	32 (26.23)	0.324
Catheterization type				
Central venous catheter (CVC)	12 (4.92)	12 (9.84)	0 (0)	<0.001
Urinary catheter	62 (25.41)	34 (27.87)	28 (22.95)	
Without catheterization	170 (69.67)	76 (62.30)	94 (77.05)	
Operative procedure based on ICD-9-CM codes				
79.31–79.32	28 (11.48)	13 (10.66)	15 (12.30)	0.049
79.35	80 (32.79)	38 (31.15)	42 (34.43)	
79.36	89 (36.48)	39 (31.97)	50 (40.98)	
Others^a^	47 (19.26)	32 (26.23)	15 (12.30)	

Values are described as *n* (%), mean ± SD, or median and interquartile range (*Q*1–*Q*3).

^a^ Included ORIF procedure in sites of multiple fractures or fractures in other sites of long bones.

### Predictors of the developed model

**Figure 1:**
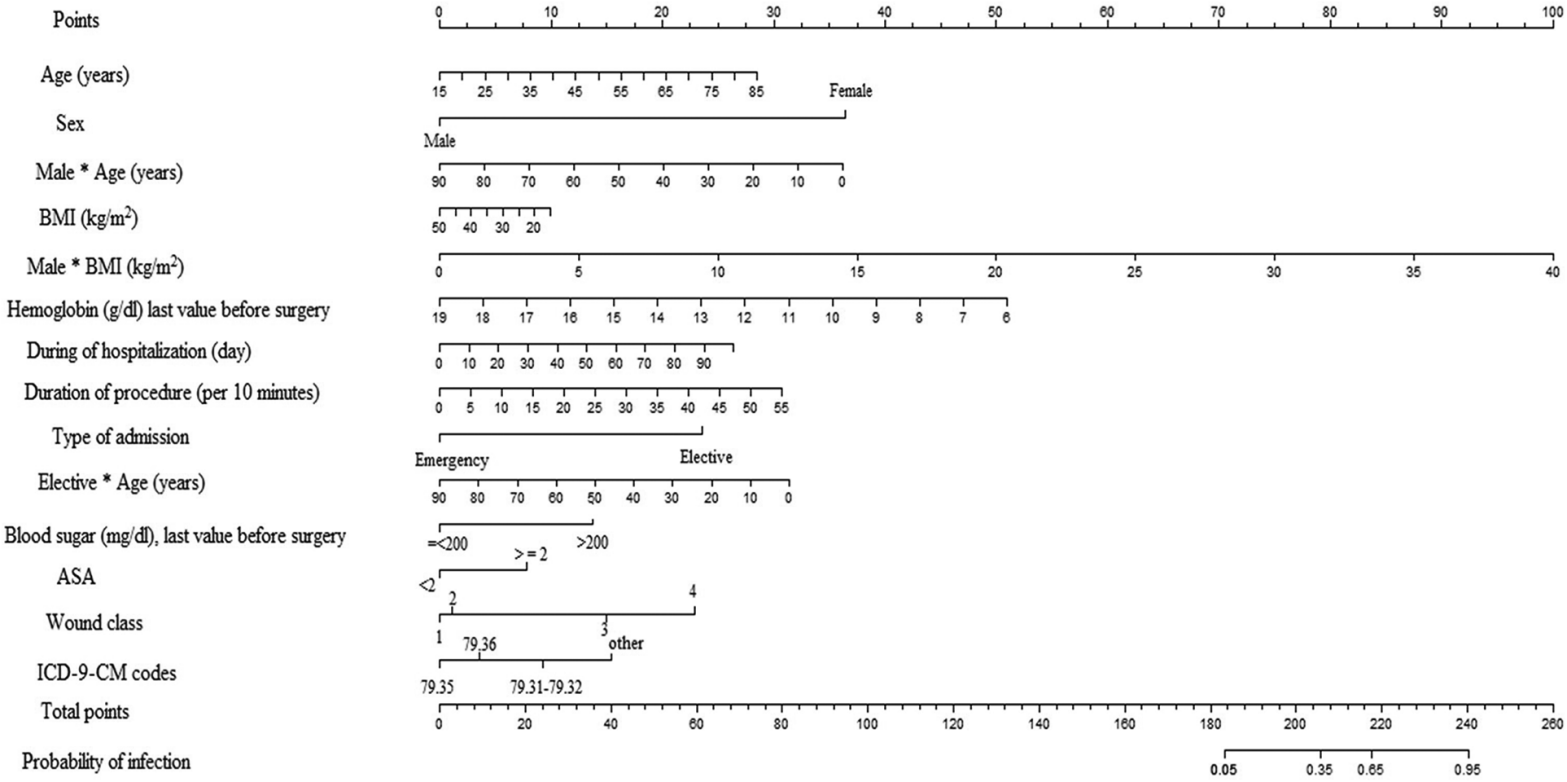
Nomogram used for the estimation of probability of SSIs following ORIF procedure based on predictors of ORIF model considering incidence outcome (SSI) of 8% in the population. Based on nomogram, each variable was given a 0–100 value that can be found to the “points” bar. The sum of these points corresponds to the “total points” bar. Total points for each patient refer to a predicted probability of SSI based on the ORIF model.

The most important predictors of the SSI after ORIF procedure were mean age of the patients, elective admission, mean duration of the procedure, blood sugar prior to surgery, ASA score, and wound class, while the mean hemoglobin level prior to surgery was the protective predictor of SSI. An interaction was also found between some variables such as gender × age and elective admission × age, and gender × BMI (kg/m^2^) (Table 2). The intercept adjusted probability of SSI (with an incidence rate of 8%) following ORIF procedure is shown as output of the model using nomogram (Figure 1).

**Table 2:** Results of univariate and multivariable logistic regression for predicting the SSIs following ORIF procedure.

Variables	Crude OR^a^ (95% CI)	*P*-value	Adjusted OR (95% CI)	Adjusted *β*	*P*-value
Age (years)	1.006 (0.99–1.02)	0.337	1.04 (1.005–1.08)	0.0423	0.024*
Sex					
Female	Reference	—	Reference	—	0.117
Male	1.19 (0.66–2.16)	0.547	0.02 (0.0002–2.58)	-3.7805	
BMI (kg/m^2^)	1.09 (1.02–1.17)	0.008*	0.97 (0.84–1.10)	-0.0295	0.665
Type of admission					
Emergency	Reference	—	Reference	—	0.008*
Elective	1.44 (0.84–2.46)	0.176	11.59 (1.85–72.40)	2.4510	
During of hospitalization (days)	1.06 (1.02–1.09)	0.0004*	1.02 (0.99–1.05)	0.0274	0.069
Hemoglobin (g/dl) last value prior to surgery	0.78 (0.69–0.89)	0.0002*	0.66 (0.54–0.81)	-0.4068	<0.0001*
Blood sugar (mg/dl), last value prior to surgery (>200 g/dl)	2.10 (0.76–5.81)	0.149	4.14 (1.05–16.34)	1.4225	0.042*
ASA score					0.029*
ASA < 2	Reference	—	Reference	—	
ASA ≥ 2	2.74 (1.61–4.65)	0.0002*	2.24 (1.08–4.67)	0.8106	
Wound class					
Class 1	Reference	—	Reference	—	—
Class 2	1.83 (0.58–5.72)	0.294	1.12 (0.28–4.42)	0.1143	0.870
Class 3	3.14 (1.59–6.21)	0.001*	4.71 (1.83–12.11)	1.5518	0.001*
Class 4	8.65 (2.84–26.33)	0.0001*	10.73 (2.50–45.96)	2.3731	0.001*
Duration of procedure (per 10 min)	1.04 (1.01–1.08)	0.007*	1.05 (1.01–1.11)	0.0580	0.014*
Operative procedure based on ICD-9-CM codes					
79.31–79.32	Reference	—	Reference	—	—
79.35	1.04 (1.01–1.08)	0.922	0.38 (0.12–1.16)	-0.9610	0.090
79.36	0.89 (0.58–5.72)	0.808	0.55 (0.19–1.58)	-0.5953	0.269
Others^b^	2.46 (0.93–6.44)	0.066	1.89 (0.58–6.09)	0.6371	0.286
Female × BMI (kg/m^2^)	—		Reference		0.006*
Male × BMI (kg/m^2^)	—	—	1.29 (1.07–1.56)	0.2596	
Female × age (years)	—		Reference		0.039*
Male × age (years)	—	—	0.95 (0.92–0.99)	-0.0418	
Emergency admission × age (years)	—		Reference	—	0.044*
Elective admission × age (years)	—	—	0.96 (0.93–0.99)	-0.0362	

Corrected intercept of model (*β*_0_) based on actual incidence of SSI following ORIF surgery = −1.65.

^a^ Odds ratio, 95% confidence interval.

^b^ Included ORIF procedure in sites of multiple fractures or fractures in other sites of long bones.

* Statistical significance, *P*-value < 0.05.

### Overall performance, validity, and calibration of the developed model

**Figure 2:**
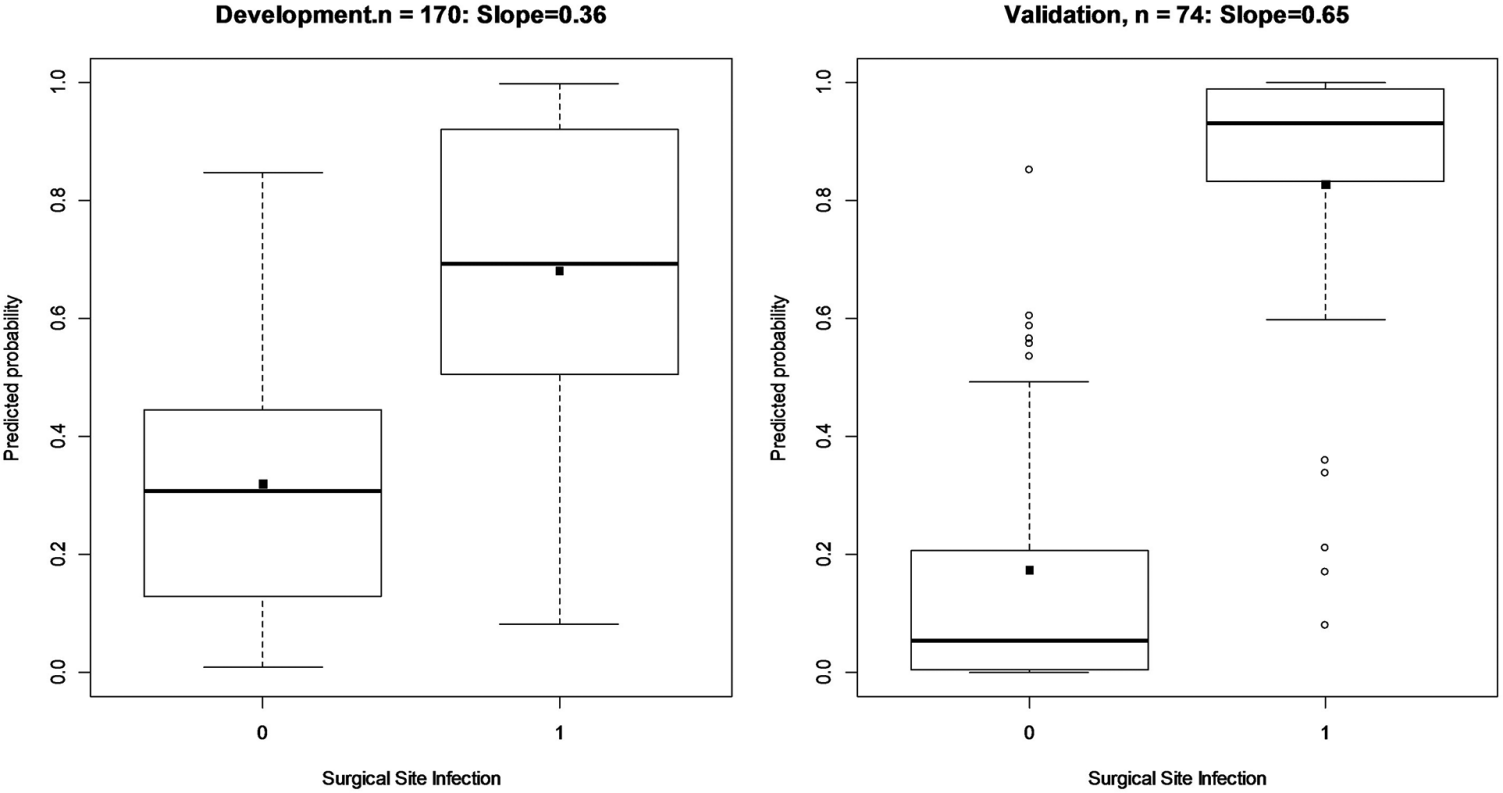
Box plot and discrimination slope of model in train (development) and test data (validation) using split-sample validation. The discrimination slopes are estimated as the difference in means of predictors for those with and without SSI following ORIF procedure. These plots show how a prediction model can discriminate those with and without the SSI.

**Figure 3:**
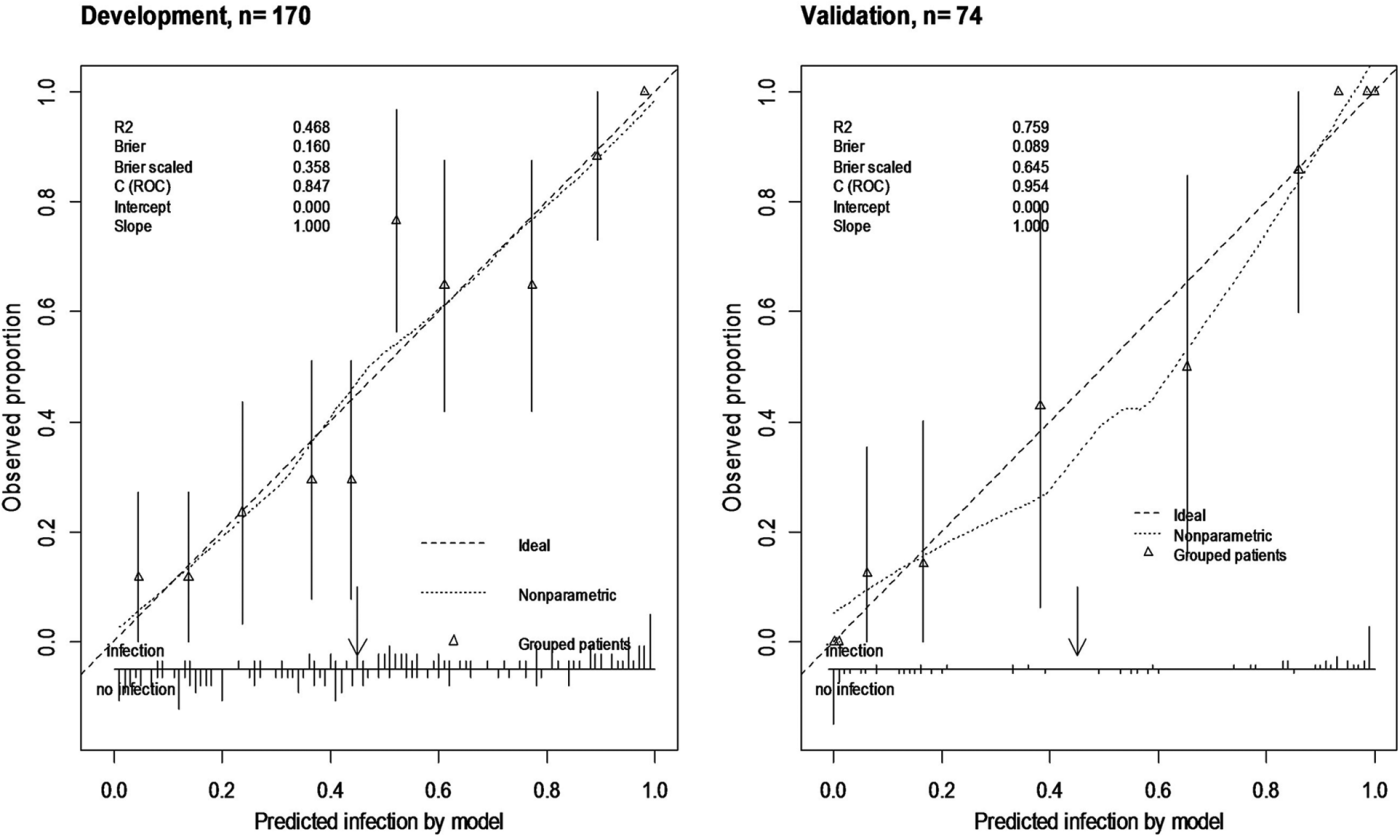
Calibration plot of ORIF model in train (development) and test data (validation) using split-sample validation. Calibration plot shows agreement of the predicted probability using model with the observed proportion of SSI (nearing of observed SSI to the 45° line) and shows clinical usefulness using the observed number of predictors above or below thresholds (arrow).

An internal validation after developing the model was assessed using a split-sample validation method (test and train approach). After performing the split-sample validation, in validation data compared with development data, the overall performance, validity, discrimination, and calibration of the developed model have improved overall (Table 3, Figure 2). At a cut-off value of 0.49 in the test (validation) data, accuracy was found to be 83%, sensitivity was 86%, and specificity was 81%. At a cut-off value of 0.49 in the test data, the AUC (95% CI) was 95% (91–99). The Brier score was 0.08 with a scaled Brier score of 0.64. The Hosmer–Lemeshow test and calibration plot showed an agreement between the predicted and observed events (*P* > 0.05) (Table 3, Figure 3).

**Table 3:** Description of performance and internal validity of the developed model for predicting the SSIs following ORIF procedure in full, train, and test data.

Indices	Full data *N* = 244	Development (train) *N* = 170	Validation (test) *N* = 70
Overall performance			
AIC^a^	276.63	200.15	78.30
*R*^2^ (Nagelkerke) (%)	44.70	46.80	75.90
Brier score	0.16	0.16	0.08
Brier scaled	0.34	0.35	0.64
Validity indices			
Optimal cut point	0.479	0.490	–
Sensitivity (%)	76.23	77.65	86.49^b^
Specificity (%)	76.23	78.82	81.08^b^
Accuracy (%)	76.23	78.23	83.78^b^
Discrimination			
AUC (C-index, 95% CI)	83.63 (78.77–88.49)	84.70 (79.0–90.40)	95.39 (91.30–99.49)
Discrimination slope (with mean incidence 8%)	0.35	0.36	0.65
Calibration			
H–L tests, *X*^2^(*P*)^c^	15.169 (0.056)	9.97 (0.266)	2.21 (0.973)

^a^ Akaike information criterion.

^b^ Based on the cut point in train data.

^c^ Hosmer–Lemeshow test.

### SIR estimation

After developing the specific model for predicting the SSI following ORIF surgery, all of the information of patients who underwent ORIF procedure during the period of March 2017 to March 2018 was extracted to estimate the SIR. In this educational hospital, a total of 350 ORIF procedures were performed within 1 year. According to the report of the infection prevention and control committee of the hospital during 1 year, only 25 patients between the ages of 18 and 85 years were infected after ORIF procedure, while the total number of predicted SSI for this population was estimated to be 37.89. Finally, the SIR for ORIF procedure was estimated to be 0.66. This estimation revealed that the observed number of SSI was lower than expected (SIR < 1).

## Discussion

One of the effective measures in preventing infection following surgery is employing multiple strategies to reduce the incidence and burden of infection. Implementing preventive measures will not prevent the infection in all of the patients; only the number of cases and the burden of infection will decrease. One of the multi-mode strategies is the application of a disease registration and surveillance system. In order to establish such a system, the patients and the disease must be defined precisely, and the effective factors in the occurrence of disease should be identified. After that, patients can be monitored through active surveillance (14). The aim of this study was to develop a prediction model for SSI and to estimate standardized infection rates after ORIF surgery in Iran. The present study is the first study in Iran to specifically evaluate and calculate the standardized infection ratio for orthopedic surgery in educational hospitals. Based on our findings, the following are the probable factors associated with occurrence of orthopedic SSIs:

Age is one of the important factors in the incidence of infection following surgery (7, 15). In our prediction model, age has a positive effect on the risk of infection. After assessing the interactive effect of age and sex, it was observed that older men had a lower chance of infection. The results of the present study are consistent with the results of the study by Yang et al. (16).

According to the results of this study, the risk of subsequent SSI was higher in elective surgeries. The result of this study is consistent with the results of the study by Talic et al. and Tan et al. (17, 18) in which patients undergoing elective surgery were at a higher risk of infection following orthopedic and general surgeries. As elective surgeries may have longer duration compared with emergency surgeries, they also may require longer hospitalization and repeat surgeries, and these factors may contribute to infection following surgery (18) However, the findings of Mukagendaneza et al. (19, 20) contradicted the results of the present study, and the incidence of infection was higher in emergency surgeries. Finally, after assessing the interactive effect of age with admission type at the hospital, it was found that the chance of infection after ORIF surgery significantly decreased with increasing age in patients with elective admission. The results of this analysis are more sensible due to the sufficient time in care and monitoring that older persons receive during elective surgeries and the small extent of injuries and fractures that older persons have compared with younger people owing to work and car accidents.

It was observed that in men with higher BMI had a higher risk of infection following ORIF, but BMI alone had no role on infection occurrence. The results of the study by Grant et al. are consistent with the results of the present study (21). Clinically, obese or overweight people, owing to the underlying diseases, poor immune response, low tissue oxygen pressure, and the wider site of injury during surgery due to more adipose tissue in these individuals, are more likely to be at risk of infection. However, according to the results of the present study, it is possible to say that the increase of BMI alone is not a cause of imminent occurrence of infection after surgery. As mentioned in the study by Waisbren et al. (22), fat tissue volume is a better predictor of SSI incidence rather than BMI. Therefore, to assess the function and effect of BMI on the incidence or possibility of infection following ORIF surgery, it is preferable to compare patients with a history of overweight or obesity with patients having normal weight or thin individuals.

An increase in the duration of the surgery has increased the chance of the infection following surgery. The results of the present study were consistent with the results of other studies (7, 23). One possible explanation for this finding is that by extending the duration of the surgery, it is possible for an incidence of more blood loss and exposure to the pathogens in the operation room, which can increase the risk of infection. Therefore, the time management of procedure is one of the major points in preventing infection after surgery.

According to the study, the type of wound in the surgical site was associated with the risk of infection; the patients who are referred to the hospital with bone damage or fractures at the surgical site, open wounds, or necrotic tissue are more prone to infection after surgery because they have lost the first line of defense of body against infection, and thus the environmental pathogens affect the tissue more compared with the patients who do not have wounds or damage. Other studies also confirmed the results of this study (7, 24).

Consistent with the results of the previous studies, the patients with ASA = 2 rank and above had higher risk of infection than those with ASA = 1 rank. As mentioned, the ASA is an index that is calculated according to the different factors that are present in the patient. Therefore, some of the probable risk factors related to the incidence of infection after surgery are measured in this index, and according to the reports of NHSN and CDC, it is one of the important indices in the prediction of infection after surgery (7, 25, 26).

According to the results of this study, one of the effective factors in lowering the risk of infection after ORIF surgery is the higher level of baseline hemoglobin. According to the literature, iron deficiency anemia is one of the major factors associated with the incidence of infection after surgery. Evidence shows that anemia and hemoglobin deficiency can cause the reduction of oxygenation and tissue blood supply, which increase the chance of infection after surgery. On the other hand, those with a history of hemoglobin deficiency and anemia are more likely to require transfusion, which is one of the most important factors of SSI after orthopedic surgery (27, 28). However, in the NHSN model, there is no report confirming the hemoglobin deficiency as a predictor variable (7). But according to the findings of various studies performed all over the world, low level of hemoglobin is an important prognostic factor after surgery, particularly orthopedic surgery.

Another important predictor of infection after surgery is the blood sugar level of the patient prior to the surgery. Various studies have confirmed the result of the present study, as diabetes has been reported to be an important factor in the incidence of infection after surgery (7, 27, 29). Because infections are more common and severe in diabetic patients than in others, common infections in healthy people may become a complication in diabetic patients. Therefore, it is recommended that diabetic patients control their blood sugar, and the physician should conduct the necessary assessments prior to surgery to determine the patient's undiagnosed diabetes and to monitor the patient's health.

In this study, after assessing the internal validity of the model, the accuracy was found to be 83% and the C-index was 95%. In comparison with the results of Mu et al. (26), the overall performance of the developed model of open reduction of fracture was 65%, while the same model with the NHSN recommended variables had a 60% overall performance. Although the proposed NHSN model is simple for application, the prediction performance of the model was poor. One of the factors contributing to the decrease in the predictive ability of the models is the lack of dedicated registration system for data related to specific surgical procedures (26). Considering that there is no dedicated registering system in Iran for collecting the factors associated with SSI and designing a better model for calculating SIR, it is possible to say that the ability of our model is preferable compared with the models in other studies worldwide. Although our statistical model may also have limitations such as possible over-fitting due to sample size, our data show that this model has a good capability. In addition, different variables and interactions were investigated in the present study in comparison with the revised NHSN model in 2018, which could be the reason for the increased functionality of the model.

According to the results of this study and the SIR index interpretation, the number of cases observed was lower than the expected cases in the hospital under study (SIR < 1). Due to the absence of the active surveillance system of NIs in Iran, it is possible that the infected patients may not referred to a hospital for treatment, and the number of cases recorded by the hospital has been under-detected and under-estimated. Therefore, it is expected that the number of cases observed is less than the actual number of cases. Also, according to the results of our previous study (30), the underestimation percentage of infection after ORIF surgery is approximately 63%.

Finally, we can conclude that the possibility of SSI after surgery is not surprising. Based on evidence and the results our investigation, environmental and individual factors, particularly antimicrobial resistance due to incorrect administering of antibiotic for self-limiting disease, affect the risk of SSIs (31). What is important is that high-risk patients are identified prior to surgery, and preventive preoperative, intraoperative, and postoperative measures for all patients are taken seriously.

According to guidelines, preventive measures such as hand hygiene of personnel and instrument sterilization, skin preparation (methicillin-resistant *Staphylococcus aureus* decolonization, hair removal, skin antiseptic), wound classification, preoperative antibiotic prophylaxis (selecting the best timing, dosing, and type of antibiotic) (32), and use of innovative device such as gentamicin bone substitutes and the application of antibacterial hydrogel and coating could be effective prophylactic tools in orthopedic surgery combined with other preventive measures (33, 34). Although implementations such as methicillin-resistant *Staphylococcus aureus* screening via swabs of the anterior nares in elective surgery is not commonly used and is institution-dependent, this implementation with other preventive measures may affect the detection of high-risk patients and, ultimately, help to reduce the risk of SSI, particularly for patients with a history of methicillin-resistant *Staphylococcus aureus* infection or a current positive screening test, as well as for patients residing in nursing homes, dorm, or prisons (32, 35).

## Limitations

One of the limitations of this case–control study is the probability of selection bias and information bias due to the retrospective design of the study and the hospital-based data. On the other hand, the study of all risk factors considered by NHSN in the Iranian hospital community, including the number of hospital beds in the room where the patients were hospitalized and the status of hospital being private or educational hospitals, was not possible. Due to the unavailability of data with regard to the number of beds in the patient's room at the time of hospitalization and the refusal of private hospitals to cooperate, the mentioned variables were ignored in this study.

## Conclusion

In general, the SIR index based on prediction models is a valid index for comparing the incidence of SSIs among hospitals. The crude incidence rate cannot be used to define the risk in a population in a period of time. Therefore, the SIR index can be used as a reliable index to estimate the occurrence of SSI, and it is used to implement infection control policies and to compare the SSI among different sections. However, in order to implement this program, the primary infrastructure and inter- and intra-section cooperation in hospitals with other health centers is necessary. On the other hand, updating existing prediction models and improving their capability should be prioritized in the surveillance system of nosocomial infection.

## Data Availability

The data analyzed in this study is subject to the following licenses/restrictions: The individual data are confidential and cannot be shared according to the ethic committee decision. Requests to access these datasets should be directed to saeedh_1999@yahoo.com.
